# Validating opioid use disorder diagnoses in administrative data: a commentary on existing evidence and future directions

**DOI:** 10.1186/s13722-023-00405-x

**Published:** 2023-08-17

**Authors:** Jeffrey F. Scherrer, Mark D. Sullivan, Marc R. LaRochelle, Richard Grucza

**Affiliations:** 1 Department of Family and Community Medicine, Saint Louis University School of Medicine, 1402 South Grand Blvd, St. Louis, MO USA; 2 Department of Psychiatry and Behavioral Neuroscience, School of Medicine, Saint Louis University, St. Louis, MO USA; 3 The AHEAD Institute, Saint Louis University School of Medicine, 1402 South Grand Blvd, St. Louis, MO USA; 4 Department of Psychiatry and Behavioral Science, University of Washington School of Medicine, 1959 NE Pacific Street, Seattle, WA 98195 USA; 5 Clinical Addiction Research and Education Unit, Section of General Internal Medicine, School of Medicine and Boston Medical Center, Boston University, Boston, MA USA

**Keywords:** Opioids, Opioid use disorder, Administrative data, Validity

## Abstract

**Background:**

A valid opioid use disorder (OUD) identification algorithm for use in administrative medical record data would enhance investigators’ ability to study consequences of OUD, OUD treatment seeking and treatment outcomes.

**Main body:**

Existing studies indicate ICD-9 and ICD-10 codes for opioid abuse and dependence do not accurately measure OUD. However, critical appraisal of existing literature suggests alternative validation methods would improve the validity of OUD identification algorithms in administrative data. Chart abstraction may not be sufficient to validate OUD, and primary data collection via structured diagnostic interviews might be an ideal gold standard.

**Conclusion and commentary:**

Generating valid OUD identification algorithms is critical for OUD research and quality measurement in real world health care settings.

## Background

### Importance of identifying a valid opioid use disorder identification algorithm

Morgan and LaRochelle’s recent commentary [1] on the development of a core outcome set for use in opioid use disorder (OUD) clinical trials highlights the challenge facing investigators who study OUD using medical record data. The authors state that addiction health services researchers have yet to develop a valid measure of OUD in electronic health records or medical claims data. They correctly note that valid OUD diagnoses in observational data are critical to evaluate quality of care and permit comparative effectiveness research. Valid OUD diagnoses in observational data offer an opportunity to leverage very large samples to study relatively rare outcomes such as overdose [1]. A valid OUD measure would also allow for identifying barriers and facilitators to medication for opioid use disorder (MOUD) treatment and MOUD therapy retention in populations that are more representative than those that are recruited to clinical trials or to prospective data collection. Common exclusion criteria such as comorbidities (e.g., schizophrenia) and lower research participation rates among minorities and underrepresentation of impoverished and rural populations limit the generalizability of clinical trials. The present mini-review is intended to critically evaluate existing efforts to estimate the validity of OUD measured by ICD-9 or ICD-10 diagnoses and offer suggestions for overcoming weak gold standards and less than ideal identification algorithms. Second, we discuss a study by Lagisetty and colleagues’ [2] in some depth because it illustrates how different factors in the medical record influence the validity of OUD diagnoses.

### ICD-9 and ICD-10 codes for abuse/dependence versus OUD

An inherent challenge to validating OUD in administrative data is the fact that ICD-9 and ICD-10 codes do not map onto OUD criteria because they define DSM-IV opioid abuse and dependence and not DSM-5 OUD. Nonetheless both codes are used as proxies for OUD in existing validation studies.

### Opioid medication contracts as a proxy for OUD

A study from the Geisinger health system observed only 2% of patients (n = 16,253) participating in a prescription opioid medication monitoring program involving opioid contracts had an ICD-9 or ICD-10 code for opioid abuse/dependence [3]. Clinical experts reviewed medical charts from 100 patients who did not adhere to their opioid contracts and 100 who did adhere and mapped medical chart information onto DSM-5 OUD criteria. Another 200 patients with two or more opioid prescriptions and not enrolled in the opioid medication monitoring program were included as controls. The investigators excluded withdrawal and tolerance because these are normal outcomes in long-term prescription opioid use. Yet they counted difficult tapers toward an OUD diagnosis. Among patients who did not adhere to their opioid contracts, 78% were classified as having moderate to severe OUD compared to 67% of those who did adhere to the contract. The 2% prevalence of OUD based on the presence of ICD-9 or ICD-10 codes is likely an underestimate. Yet the 67–78% of patients classified as having moderate to severe OUD is remarkably high. This could be partly due to the medical abstractors not being blinded to patients’ OUD diagnosis. In addition, the liberal use of indicators for OUD could increase false positives. For instance, the authors counted “vocational interference owing to drug use or pain” and “difficult opioid tapers” toward an OUD diagnosis. While these symptoms are concerning, they do not clearly match OUD symptoms. Together this highlights the problem of using ICD codes alone to identify OUD cases. Although the authors conclude that opioid contract violation is a good proxy for OUD, this study was not a classic validation study in that it did not involve comparing agreement with a gold standard and did not compute the sensitivity and specificity of OUD ICD-9 and ICD-10 codes.

McNeely and colleagues [4] recently observed excellent agreement between EHR cases of OUD subsequently documented in Medicaid claims. Specifically, ICD-10 codes were used to identify patients with OUD who received MOUD while in hospital. Among these patients, 84.2% had the diagnosis in medical claims. The good sensitivity from this study may be driven by the hospital setting. Hospital based OUD treatment likely captures more severe OUD and leaves little room for diagnostic error. Last, this study was not designed to validate OUD diagnoses in the medical record but it does indicate OUD diagnoses in Medicaid claims are sufficiently valid to utilize in epidemiological and health services research. However, a valid OUD identification algorithm is still needed for EHR data and private health insurance claims.

### Comparison of ICD-9 and ICD-10 diagnoses to chart abstraction

Howell and colleagues [5] identified 90 cases of incident OUD in Veterans Health Affairs (VHA) health care. Cases were defined as at least one visit with an ICD-10 code for opioid abuse or dependence. An expert panel evaluated the accuracy of diagnoses based on corroborating clinical documentation/notes 30 days prior and 90 days after index diagnoses. This resulted in 29% of ICD-9 and ICD-10 diagnoses being coded as likely inaccurate. The poor agreement could be due to the brief period used to abstract clinical notes because the DSM criteria allows for a 12 month period for OUD symptoms to cluster. A separate study involving expert review of medical records from non-VHA health care settings revealed ICD codes had 59% sensitivity (true positive rate) and 93% specificity (true negative rate) compared to expert chart abstraction [6]. Unfortunately this study did not include sufficient detail about the identification algorithm to fully understand potential factors contributing to only moderate sensitivity. Typically, observational designs use identification algorithms that maximize sensitivity (i.e., true positives) which has led to many published algorithms that require repeated clinic encounters for the same diagnosis within a fixed period of time.

While evidence of OUD symptoms can be derived from reviewing clinician notes, there are limitations of this approach. Clinician notes are not always comprehensive and may not contain details about OUD symptoms. Using expert review of medical notes to create a gold standard diagnosis is limited by the fact that interpreting charts is dependent upon subjective decisions [5]. For example, a medical chart may contain information about a positive urine drug screen (UDS) for a non-prescribed opioid among patients on long-term opioid therapy (LTOT). This may suggest non-prescribed use but may be a false positive result or use that does not amount to a use disorder.

The study by Lagisetty and colleagues [2] utilized VHA administrative medical record data and ICD codes for opioid use, abuse and dependence to create an OUD identification algorithm. These investigators defined OUD cases by requiring at least two diagnoses on separate days between 2012 and 2017. The authors then randomly sampled 520 charts for abstraction from patients who had annual VHA encounters for two years without ICD-9 or ICD-10 codes for opioid abuse or dependence and then had a new and possibly incident ICD code for opioid use, abuse or dependence. Charts were abstracted for the month prior and 3 months after the first of the two diagnostic codes. Only 58% of patients with diagnostic codes for use, abuse or dependence could be validated as OUD by manual chart abstraction. However, an 80% agreement would be obtained if cases with insufficient information were removed from analyses and patients with high likelihood of OUD and those with aberrant opioid use were combined. This assumes aberrant opioid use indicates using more than prescribed, diverting opioids or other non-medical use. Yet it is possible that patients would look like they were diverting medication if they did not consume the amount prescribed. The number of charts lacking data about OUD also raises the possibility that insufficient look-back time was used to search charts to support OUD diagnoses. The authors conclude that opioid abuse and dependence diagnoses are not a valid proxy for OUD and thus using them for large cohort studies of OUD outcomes and treatment seeking would not be appropriate. However, we believe the literature has not considered alternative OUD identification algorithms and it would be premature to assume OUD diagnoses derived from ICD codes are not valid.

### The importance of repeat OUD diagnoses within a narrow period

Another reason we caution against accepting the conclusion that medical record opioid abuse and dependence diagnoses have poor validity is Lagisetty and colleagues [2] use of a 4 month chart abstraction period. This is a substantially narrower time period than the DSM-5 requires which is 1-year for symptoms to co-occur and is a short period for validation of diagnoses that could have occurred five years apart. While we agree with Lagisetty and colleagues’ approach of using two diagnostic codes to define OUD, we believe that allowing a 5 year window for two diagnoses to occur may have reduced specificity. Allowing a long temporal window to select cases for validation may have selected individuals who met criteria for OUD at some point during the selection window, but not necessarily during the relatively short window of time (4 months) used for validation via manual chart abstraction.

There is also evidence that restricting the timeframe in which diagnoses occur can improve validity which means a single diagnoses for opioid abuse or dependence in 1 year and a second 5 years later would be more likely to be a false positive compared to an approach that requires two diagnoses in a year or less. For instance, the validity of depression diagnoses is excellent when depression is defined as 2 or more outpatient or 1 inpatient ICD-9 diagnoses in a 12 month period [7]. Two diagnostic codes for posttraumatic stress disorder (PTSD) in 4 months had an 82% positive predictive value when compared to a gold standard PTSD Checklist (PCL) score ≥ 50 [8].

### Types of opioid use disorder

Lagisetty et al. [2] observed that 19% of patients with diagnostic codes for opioid abuse/dependence only had prescription opioid use for pain documented in the medical chart. If these patients had long-term opioid therapy (LTOT), defined as > 90 days, and used prescription opioids daily or near daily, they most likely developed physiological dependence. According to DSM-5, this should not be counted toward an OUD diagnosis, but it is possible that providers use the opioid dependence diagnoses to identify physiological dependence rather than disordered opioid use.

Future validation studies should consider the potential information gained by validating prescription OUD (POUD) separately from other OUD because patients with POUD can also be offered MOUD either for risk reduction as part of continued pain management or following unsuccessful taper. As noted by Hasin and colleagues [9], we need to understand risk factors and outcomes of POUD because prescribing rates, although in decline, remain much higher than prior to the opioid epidemic. It is possible that MOUD outcomes will differ in patients without comorbid substance disorders who develop POUD during opioid therapy [10]. If we can distinguish two valid OUD phenotypes, OUD and POUD, health services research and outcomes research can begin to determine if pathways to treatment and therapeutic outcomes differ between these two patient populations.

Whether DSM-5 criteria should be applied to patients using prescription opioids for pain is an on-going debate. The Psychiatric Research Interview for Substance and Mental Disorders, DSM-5, opioid version, (PRISM-5-OP) demonstrated improved validity when adapted for patients receiving opioid therapy for pain [9]. This highlights the need to validate administrative medical record algorithms that distinguish between POUD related to pain treatment vs. addictive behaviors, using opioids for non-medical purposes to cope with stress or taking opioids to get high. However, as Sullivan and Ballantyne [11] note, this study assumes LTOT for pain is safe and effective and withdrawal and tolerance are benign. This has proven not to be the case and POUD may begin with hyperkatifeia where persistent opioid use is an attempt to relieve negative mood or interdose withdrawal.

Studies validating OUD diagnoses in administrative data have yet to separately validate POUD and other forms of OUD. Identifying POUD in administrative data is challenging because it requires data that distinguishes between patients with POUD with no evidence of illicit (heroin, fentanyl) opioid use versus patients with POUD who have comorbid substance use disorders and or opioid misuse. Boundaries between LTOT, opioid misuse, and POUD are fuzzy. What appears to be simple LTOT may have evolved into POUD. Further complicating the effort is that OUD and POUD may not be orthogonal and instead could represent a continuum with patients potentially fitting into more than one OUD group.

## Conclusion and commentary

Revisiting the observation period in Lagisetty and colleagues [2] analyses and looking back at least 1 year for indications of OUD and using narrower time limits on their two diagnoses requirement may enhance the validity of OUD diagnosis. If results change, this would emphasize the importance of the reference standard in evaluating the validity of case identification algorithms. Given the number of charts lacking useful information, chart abstraction may generate more fruitful findings using an even longer, 2 year look back period. In addition, it would be informative to compare different identification algorithms for OUD to be validated against chart abstraction. One algorithm could require a single-diagnosis and a second require 2 diagnoses in a 12 month period and a third require 2-diagnoses in a 2 year period.

We propose starting by sampling patients from administrative medical record data who have two or more ICD-10 codes for opioid abuse or dependence in the same 12 month period. From this sample, potential OUD cases would be selected by requiring no current prescription opioid use. Potential POUD cases would require patients to have past or current LTOT and one or more of the following: comorbid substance use disorders, repeated early or late opioid fills, and/or comorbid externalizing disorders. This is a reasonable starting point for sampling and chart abstraction of physician notes will determine if each OUD type can be distinguished from one another. Algorithms that add confirmatory diagnoses such as opioid overdose or include prescriptions for OUD should be evaluated to determine if they improve validity. Of course, each identification algorithm has advantages and disadvantages. For instance, by requiring LTOT co-occurring with comorbid substance use disorders, the sample will be biased toward patients who have likely sought treatment and received a substance use disorder diagnosis. If we sampled only on LTOT, many intermittent prescription opioid users will be captured and these patients are less likely to have physiological dependence compared to daily users. This could lead to more classification errors when identifying OUD cases. Ideally, when OUD is a primary exposure or outcome, the algorithm needs to maximize specificity and sensitivity. However, it is common to see a single diagnosis for OUD when treated as a covariate. This is a reasonable choice, particularly for time varying covariates, because a single diagnosis vs. two diagnoses within a restricted time frame allows for increased ability to detect change over time.

Administrative data includes both medical claims and medical records. Compared to claims data, medical records contain much more information for case identification such as provider notes and multiple diagnoses, not just the condition for which a medical claim was paid. Medical record data should come from a healthcare system in which patients are frequently retained over many years. Thus, the VHA is an ideal starting point. After identifying probable cases of OUD and POUD, structured diagnostic interviews would be administered to these patients to establish gold standard diagnoses. Healthcare systems, provider and geographic factors can bias the accuracy of OUD diagnoses. For instance, healthcare settings that use annual substance use disorder screenings may contain more accurate case identification. Providers with more training in OUD should be expected to generate fewer false negatives and false positives. Thus, factors contributing to validity in one healthcare system region may not be the same elsewhere depending on system, provider and geographic variation.

An ideal validation study should consider different groups of OUD. Ideally, validation should classify patients OUD status using a structured diagnostic interview as a gold standard. Determining which identification algorithms have superior validity would establish the best approach to define OUD and POUD in administrative medical record data. Valid identification algorithms likely exist, and when found, can be utilized by the large number of investigators using big medical record data to study these patient populations.

## Data Availability

Not applicable.
